# Critical Functions of Histone Deacetylases (HDACs) in Modulating Inflammation Associated with Cardiovascular Diseases

**DOI:** 10.3390/pathophysiology29030038

**Published:** 2022-08-22

**Authors:** Supaporn Kulthinee, Naohiro Yano, Shougang Zhuang, Lijiang Wang, Ting C. Zhao

**Affiliations:** 1 Cardiovascular and Metabolism Laboratories, Department of Surgery and Plastic Surgery, Rhode Island Hospital, Warren Alpert Medical School of Brown University, Providence, RI 02903, USA; 2 Department of Medicine, Rhode Island Hospital, Brown University, Providence, RI 02903, USA; 3 Department of Surgery, Boston University Medical School, Boston, MA 02118, USA

**Keywords:** HDACs, cytokines, inflammation, cardiovascular diseases

## Abstract

Histone deacetylases (HDACs) are a superfamily of enzymes that catalyze the removal of acetyl functional groups from lysine residues of histone and non-histone proteins. There are 18 mammalian HDACs, which are classified into four classes based on the primary homology with yeast HDACs. Among these groups, Class I and II HDACs play a major role in lysine deacetylation of the N-terminal histone tails. In mammals, HDACs play a pivotal role in the regulation of gene transcription, cell growth, survival, and proliferation. HDACs regulate the expression of inflammatory genes, as evidenced by the potent anti-inflammatory activity of pan-HDAC inhibitors, which were implicated in several pathophysiologic states in the inflammation process. However, it is unclear how each of the 18 HDAC proteins specifically contributes to the inflammatory gene expression. It is firmly established that inflammation and its inability to converge are central mechanisms in the pathogenesis of several cardiovascular diseases (CVDs). Emerging evidence supports the hypothesis that several different pro-inflammatory cytokines regulated by HDACs are associated with various CVDs. Based on this hypothesis, the potential for the treatment of CVDs with HDAC inhibitors has recently begun to attract attention. In this review, we will briefly discuss (1) pathophysiology of inflammation in cardiovascular disease, (2) the function of HDACs in the regulation of atherosclerosis and cardiovascular diseases, and (3) the possible therapeutic implications of HDAC inhibitors in cardiovascular diseases. Recent studies reveal that histone deacetylase contributes critically to mediating the pathophysiology of inflammation in cardiovascular disease. HDACs are also recognized as one of the major mechanisms in the regulation of inflammation and cardiovascular function. HDACs show promise in developing potential therapeutic implications of HDAC inhibitors in cardiovascular and inflammatory diseases.

## 1. Introduction

Histone Deacetylases (HDACs) are a superfamily of enzymes that remove an acetyl group from lysine residues of the N-terminal regions of histone and non-histone proteins [1]. Eighteen HDACs have been identified in mammalian cells, which can be classified into four main classes (Classes I, II, III, and IV). Of these groups, Class I and II HDACs play a primary role in lysine deacetylation [2]. Several studies have now demonstrated that aberrant hyper HDAC activity and histone hypoacetylation are associated with the abnormalities in the transcription of key genes and the development of diverse diseases. Hence, the inhibition of HDAC activity has come into the limelight as a strategy for treating cancers and a wide variety of diseases.

HDAC inhibitors, the most common means of inhibiting HDAC activity, are chemicals that inhibit HDACs. HDAC inhibitors have long been used in psychiatry and neurology as mood stabilizers and antiepileptic drugs [3]. In recent years, they have been investigated as a treatment for cancer, parasitic infections, inflammatory diseases, and cardiovascular diseases [4,5,6,7]. Thus, research on the disease suppression mechanism of HDAC inhibitors has intensified in recent years, as it should. The availability of specific HDAC inhibitors such as trichostatin A (TSA) and suberonyl iridohydroxamic acid (SAHA) has greatly helped to advance research on the role of HDACs in gene regulation. Among the various studies on HDAC inhibitors, the effects on the immune response and inflammation have been a major topic, and there are extensive reports of relevant research. For example, the treatment of splenocytes isolated from MRL-lpr/lpr lupus mice with TSA or SAHA resulted in the suppression of transcriptions and protein levels of inflammatory cytokines such as interleukin (IL)-6, IL-12 and interferon (IFN)-γ [8]. In addition, intraperitoneal injection of SAHA downregulated IL-6, tumor necrosis factor (TNF)-α, and chemokine CCL2 in the colonic tissue of the dextran sulfate sodium (DSS)-induced mouth colitis model [6]. Moreover, intraperitoneal injection of SAHA to lipopolysaccharide (LPS)-treated ddY mice attenuated peripheral inflammation-induced cognitive dysfunction [6]. 

Inflammation is a defense mechanism of the immune system’s response to harmful stimuli that is vital to health. The inflammation process acts by removing injurious stimuli and initiating the healing process. However, incomplete healing of acute inflammation may become chronic inflammation, which contributes to serious chronic inflammatory diseases [9]. Recently, the role of inflammation in the development of atherosclerosis and consequent cardiovascular disease is gaining increasing recognition [10,11,12]. Accordingly, the potential of an HDAC inhibitor, with its anti-inflammatory properties, as a therapeutic agent for cardiovascular disease has also been addressed [13,14]. 

In this review, the involvement of HDACs in the development of cardiovascular diseases (CVDs) is outlined based on recent findings. In addition to that, perspectives on the potential of HDAC inhibitors as a therapeutic agent for CVD will also be discussed.

## 2. Classes of HDACs

**Figure 1:**
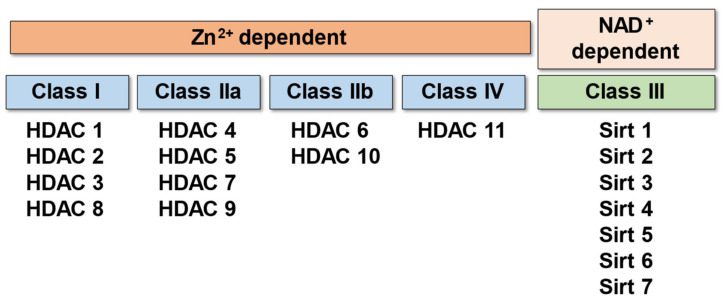
Classification of HDACs.

HDACs are a superfamily of enzymes that remove an acetyl group from the N-terminal regions of histone proteins allowing the histones to wrap the DNA more tightly [13]. Therefore, deacetylation by HDACs results in decreased gene transcription, and its action is opposite to that of histone acetyltransferases (HATs). So far, 18 HDACs have been identified in mammalian cells. Those 18 mammalian HDACs have been classified into four distinct classes (classes I, IIa, IIb, III, and IV) according to the systematic analysis of sequence homology, enzymatic activity, domain structure, and functional similarity (Figure 1).

Class I is homologous to yeast Rpd3 and includes HDACs 1, 2, 3, and 8. Class I HDACs are ubiquitously expressed in mammalian tissues and form complexes with protein subunits such as Sin3 and N-Cor, which promote histone deacetylation and repress gene transcription [15,16].

Class II is a homologue of yeast HDA1, whose N-terminus has conserved domains attributed to protein–protein interactions. The six HDACs in this class are further classified into two subclasses according to the presence or absence of myocyte enhancer factor (MEF) binding sites (MEF-BS): Class IIa (MEF-BS+) and Class Iib (MEF-BS-). Class II HDACs are expressed specifically in organs such as skeletal muscle, the heart, and the brain. Unlike Class I HDAC, which localizes predominantly to the nucleus, Class II is known to move between the nucleus and cytoplasm, suggesting its function as a stimulus transducer [17,18].

Class III is a homologue of the silent information regulator 2 (Sir2) family of proteins, which includes seven variants, SIRT1 through SIRT7 [19]. Among them, SIRT1 and 7 are known to be involved in cardiac development and have protective functions against stress- and age-related cardiac dysfunction [20]. Sirtuins require NAD+ for deacetylation and play an important role in transcriptional repressor function [21].

HDAC11, the only member of Class IV, is functionally similar to Class I and Class II, but has very limited amino acid sequence homology and cannot be classified into the other three classes [22]. The expression of HDAC 11 has been shown in multiple organs such as the kidney, brain, heart, and skeleton muscle [19]. Of note, classes I, II, and IV are zinc-dependent enzymes while class III is NAD+-dependent.

## 3. HDACs and Inflammations

It is becoming increasingly clear that HDACs are fundamentally involved in the regulation of genes that are widely expressed during inflammation, suggesting that HDACs directly influence the inflammatory process. For example, treatment of splenocytes isolated from MRL-lpr/lpr lupus mice with the HDAC inhibitor TSA or SAHA resulted in the suppression of transcriptions and protein levels of decreased mRNA expression of inflammatory cytokines such as IL-6, IL-12, and IFN-γ [8]. The proinflammatory effects specific to each HDAC class are also gradually being revealed as follows.

### 3.1. Class I HDACs

HDAC 1 levels are elevated in synovial fluid of rheumatoid arthritis (RA) patients, and HDAC 1 levels correlate with the expression of TNF-α in RA synovial tissues [23]. The levels of proinflammatory cytokines IL-17 and IL-6 in serum were significantly reduced in T cell-specific conditional HDAC 1-KO mice [24]. The HDAC 1-KO mice showed resistance to the development of collagen-induced arthritis (CIA), but the antibody response to type II collagen was not inhibited, indicating that T cell-mediated activation of B cells was not impaired [24]. These data suggest that HDAC 1 has an enhancing effect on T-cell mediated immune response. 

The expression levels of HDAC 2 were significantly decreased in lung tissues of chronic obstructive pulmonary disease (COPD) patients [25]. Protein expressions of HDAC 2 in the airways were decreased in asthmatic patients [26]. The restoration of HDAC 2 peripheral blood mononuclear cells from COPD patients attenuated corticosteroid resistance of the patients [27]. These findings suggest that HDAC 2 is involved in the maintenance of an inflammatory response in chronic inflammatory diseases.

HDAC 3 induces monocyte migration to inflammatory sites and promotes cytokine production by macrophages at the inflammatory sites. Conversely, LPS-responsive cytokine production is reduced in macrophages with deficient HDAC 3 [28]. The mechanisms by which HDAC 3 promotes monocyte infiltration into inflammatory sites have been reported, including increased expression of MCP-1 and VCAM-1 [29,30]. On the other hand, it has been reported that the production of proinflammatory cytokines by monocytes is increased when HDAC 3 function is reduced or absent [31,32]. Therefore, the relationship between inflammation and HDAC3 is still controversial.

It has been reported that as the severity of COPD increases, the expression of HDAC 8 in the trachea decreases [33]. HDAC 8 is upregulated late in human osteoclastogenesis in vitro, and this is consistent with the experimental fact that HDAC 8 is essential for normal craniogenesis [34].

### 3.2. Class IIa HDACs

HDAC 4 has been shown to play a role in repressing the transcription of IL-5, an important mediator of inflammatory processes in allergic diseases [35]. HDAC 4 was also shown to promote ROS-dependent vascular inflammation, possibly via the expression of VCAM-1 [36]. Furthermore, the expression of HIF-1α was downregulated in cells silenced with HDAC 4 by siRNA [37]. HIF-1a is a key transcription factor in the pathogenesis of various inflammatory diseases including RA, suggesting a link between HDAC 4 and inflammatory diseases [38].

HDAC 5 is known to be involved in inflammation via the activation of monocytes and macrophages [39]. HDAC 5 has also been shown to promote immune cell adhesion to inflammatory lesions [40]. This is consistent with the identification of the HDAC 5 gene as a locus that influences bone mineral density [41]. In fact, HDAC 5 expression is known to be markedly upregulated in human osteoclasts during the late developmental stage [42]. HDAC 5 expression was increased in RANKL-induced osteoclasts in vitro, and the high levels of RANKL have been shown to be associated with the pathogenesis of bone loss in inflammatory diseases such as periodontitis and RA [43,44].

CD8/CD4 double-positive platelets are known to express HDAC 7, which may regulate cell survival and T cell receptor signaling [45]. HDAC 7 may also be involved in the expression of HIF-1α [46]. In relation to bone, it has been shown that suppressing HDAC 7 promotes osteoclast differentiation. Thus, it is suggested that HDAC 7 inhibits osteoclast formation [47]. Interestingly, the activity of nuclear HDAC 7 has been shown to correlate with that of HDAC 3 [48]. This indicates the complexity of the functional relationships among the HDACs enzymes and the difficulty in identifying the function of specific HDACs in inflammation.

Phosphorylation of Nf-κb subunit p65 by Tnf-α is reduced in HDAC 9-deficient macrophages. This finding suggests that epigenetic regulation of HDAC 9 is involved in inflammation and immune responses [49]. The involvement of HDAC 9 in the inflammatory response was confirmed by LPS treatment on cultured cells. When HDAC 9 is deficient, CD4+ T cells stimulated in vitro secrete significantly less Il-2 and Il-12 and produce modestly less IFN-γ, while ex vivo Il-4 secretion is increased [50]. This suggests that HDAC 9 may act as an epigenetic switch in effector T cell-mediated systemic autoimmunity. Another study found that in the absence of HDAC 9, activation of different inflammatory signaling pathways, such as Erks, p38, Jnk, Nf-kβ, and Ikbα, was suppressed [51]. Thus, the contribution of HDAC 9 to inflammation is plausible, but the molecular mechanism of its action requires further investigation.

### 3.3. Class IIb HDACs

HDAC 6 is known to promote Inflammatory tolerance induced by LPS in astrocytes [52]. Other studies have shown that HDAC 6 is involved in the regulation of T regulatory (Treg) cells [53]. Inhibition of HDAC 6 with a specific HDAC inhibitor promotes Treg suppressive activity in models of inflammation and autoimmunity such as experimental colitis and cardiac allograft rejection [53]. However, other studies have also shown that HDAC-deficient macrophages exhibit normal inflammatory responses, suggesting that other HDACs may compensate for HDAC 6 function in macrophage inflammatory responses [54].

HDAC 10 is highly linked to the local expression of IL-1β in vivo and in vitro. HDAC 10 knockdown alleviated the activation of synovium-derived mesenchymal stem cells (SMSCs) by IL-1β and inhibited activation of the NF-κB pathway. In contrast, the overexpression of HDAC 10 promoted IL-6 and IL-8 expression and IL-1β-mediated activation of the NF-κB pathway [55].

### 3.4. Class IV HDAC

Obesity and metabolic syndrome are usually accompanied by chronic low-level inflammation in the background [56]. HDAC 11 plays an important regulatory role in metabolic inflammatory processes. HDAC 11 suppresses metabolic inflammation primarily by regulating IL-10 secretion by antigen-presenting cells (APCs) [57]. Upon inhibition of HDAC 11 function, IL-10 secretion by macrophages is increased. On the other hand, the overexpression of HDAC 11 suppresses IL-10 production. This is caused by the fact that in activated macrophages, HDAC 11 is recruited to the distal end of the IL-10 promoter by a delayed kinetic effect and HDAC 11 also directly interacts with the distal region of the IL-10 promoter [58]. In addition, imipramine, a drug in use for the treatment of depression and nocturnal enuresis in children, upregulates HDAC 11-mediated inhibition of IL-10 production and upregulation of IL-12 by Leishmania donovani-infected mouse macrophages [59].

### 3.5. Class III HDACs

The association between Class III HDACs and inflammation is described less extensively than for other HDACs. However, still, among the few, there are several reports on Sirt1-mediated mechanisms of suppression of inflammation [60,61,62].

**Figure 2:**
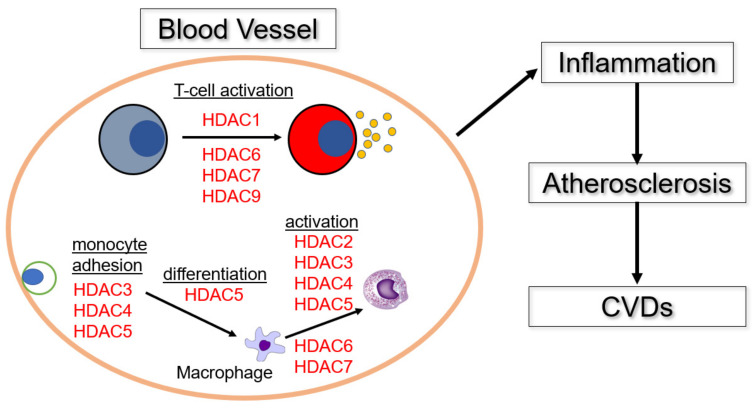
The roles of HDACs in inflammation and development of CVDs. HDAC3, 4, and 5 promote migration and adhesion of monocytes to inflammatory sites [29,30,63,64], followed by induction of macrophage differentiation by HDAC5 [65]. The differentiated macrophages are activated by HDAC 2, 3, 4, 5, 6, and 7 [28,32,40,66,67]. And HDAC 6, 7, 8, and 9 promote T cell activation at inflammatory sites [53,54,68,69].

The involvement of HDACs in the immune response and induction of inflammation in blood vessels is summarized in Figure 2.

## 4. Pathophysiology of Inflammation in Cardiovascular Disease

Generally, atherogenic changes precede the onset of cardiovascular disease [10,11,12]. The process of atheroma formation involves inflammation induced by both the innate and adaptive immune systems [70]. The innate immune system is the mechanism of immediate and nonspecific host defense against an unknown antigen [71]. Chemokines secreted by non-sensitized immunocompetent cells in response to an encounter with an unfamiliar antigen induce mononuclear phagocytes to migrate to the vascular intima via leukocyte adhesion molecules [10]. These monocytes mature into macrophages in the vascular intima and further differentiate into foam cells, from which plaque is formed. The adaptive immune system presents recognized antigens to B cells, stimulating antibody production, which, in turn, contributes to lesion extension, smooth muscle proliferation, increased platelet reactivity, and thrombo-occlusion [71]. Another important component of the innate immune system in the cardiovascular system is the Nod-like receptor protein 3 (NLRP3) inflammasome [72]. The NLRP3 inflammasome is an intracellular sensor that detects various microbial motifs, endogenous danger signals such as cholesterol crystals, and environmental aggressors. [11,71]. NLRP3 activation is thought to involve S100A12, a member of the S100 family of inflammatory proteins [73]. S100A12 is a proinflammatory factor that causes diabetes-induced activation of retinal microglia by activating NLRP3 in vitro and in vivo. S100A12 induced the expression of NLRP3 with microRNA in an miR-30A-dependent manner [73]. HDACs may be indirectly involved in NLRP3 activation by regulating miR-30A expression [73,74]. Upon detection of these danger signals, the NLRP3 inflammasome activates the caspase-1 enzyme. Caspase-1 then cleaves pro-IL-1β and pro-IL-18 into active IL-1β and IL-18 [75]. Both IL-1β and IL-18 contribute directly to plaque growth and expansion and induce local production of IL-6 [76]. IL-6 is a highly active cytokine secreted by activated leukocytes and vascular smooth muscle. IL-6, by inducing migration of inflammatory cells, promotes low-density lipoprotein (LDL) uptake and oxidation by lipid-depositing macrophages, stimulating smooth muscle cell proliferation and enhancing the prothrombotic effects of platelets, thereby promoting atherosclerosis [77]. 

As clinical evidence supporting the relationship between inflammation and CVDs, patients with systemic chronic inflammatory diseases tend to have an increased risk of developing CVDs. The meta-analysis in chronic inflammation indicated that the risk of developing CVDs is increased by 48% in patients with rheumatoid arthritis compared to the general population [78]. Moreover, in rheumatic patients, the risk of myocardial infarction and stroke was higher by 68% and 41%, respectively [78]. Systemic inflammatory autoimmune diseases such as systemic lupus erythematosus (SLE) also increased the risk of CVD and stroke [79]. Furthermore, patients with psoriasis have an increased risk of developing cardiomyopathy and heart failure [80]. 

For the etiological association between inflammation and CVDs, emerging evidence supports the hypothesis that overactivity of various inflammatory cytokines, independently of other factors, increases the risk of developing CVDs. For example, a 1-SD higher baseline serum level for each of IL-6, IL-18, and TNF-α is associated with a 10–25% higher risk of non-fatal myocardial infarction (MI) or coronary heart disease (CHD) death [81]. In the Cardiovascular Risk Reduction Study (CANTOS), a randomized double-blind trial of the anti-inflammatory therapy targeting the interleukin-1β innate immunity pathway, patients receiving canakinumab, a therapeutic monoclonal antibody targeting interleukin-1β, had a lower incidence of recurrent cardiovascular events than those receiving placebo [82]. In addition, the interleukin-1β inhibitor reduced hospitalization for heart failure (HHF) and the composite of HHF or heart failure-related mortality in patients with prior myocardial infarction and elevations in the high-sensitivity C-reactive protein [83]. These studies further support the etiological linkage between inflammation and the development of CVDs.

## 5. The Function of HDACs in Regulation of Atherosclerosis and Cardiovascular Diseases

Altered expression of HDACs modulates cellular function by affecting the transcription of diverse proinflammatory genes that regulate important cellular events in cardiomyocytes, vascular endothelial cells, and vascular smooth muscle cells. Thus, the disruption of HDAC expression may be directly related to the development of CVDs. The relationship between HDACs and the pathogenesis of various CVDs is described below.

### 5.1. Cardiac Hypertrophy

Cardiac hypertrophy may be the most studied cardiovascular morbidity in the context of HDAC and its pathogenesis. Cardiac hypertrophy is a type of adaptation to hemodynamic changes caused by peripheral tissues and underlying conditions such as physical overload, chronic hypertension, myocardial ischemia, myocarditis, cardiomyopathy, and various other cardiac conditions [84]. While the initial adaptation may be physiological, cardiac hypertrophy is the beginning of the global remodeling of the heart. The role of HDACs in cardiac hypertrophy has been extensively studied by many research groups. Both Class I and Class IIa HDACs are involved in the development of cardiac hypertrophy, but their roles are quite opposite. Cardiac-specific overexpression of HDAC 2 induces cardiac hypertrophy [85,86]. Thus, HDAC2 clearly causes cardiac hypertrophy, but the levels of the HDAC 2 protein are not altered in the process. The intrinsic activity of HDAC 4 is increased by activated CK2α1 in response to hypertrophic stimuli [87]. As for Class I HDACs, no clear evidence has been found to date that Class I HDACs other than HDAC 2 are involved in cardiac hypertrophy. However, HDAC 3 may show the transient proliferative potential of cardiomyocytes in the perinatal period [88]. Class IIa HDACs, on the other hand, suppress cardiac hypertrophy. HDACs 4 and 5 have been shown to function as endogenous repressors of genes involved in pathological cardiac hypertrophy by traveling between the nucleus and cytoplasm [89]. The anti-cardiac hypertrophic effects of Class Iia HDACs occur through direct binding to the myocyte enhancer factor 2 (MEF2) transcription factor, a hypertrophy-promoting factor, or through indirect binding to other hypertrophic signaling factors [89,90]. Hypertrophic stimulation attenuates the hypertrophic inhibitory effect of Class Iia HDACs by phosphorylating them and inducing their nuclear export [89,91]. In cardiomyocytes, in response to pressure overload, the evidence described that CaMKIIδ induces the nuclear export of HDAC 4 through phosphorylation, thereby releasing MEF2 [92]. Redistribution of class IIa HDACs induces reactivation of arrested-fatal gene programs regulated by MEF2, leading to cardiac hypertrophy. In adult ventricular cardiomyocytes, the hypertrophic enhancer endothelin-1 causes nuclear membrane Ca^2+^ release through the activation of inositol 1-4,5-trisphosphate receptors and induces phosphorylation and nuclear export of HDAC 5 [93]. Protein kinase D1 has also been identified as one of the Class IIa HDAC kinases important in the development of pressure-induced cardiac hypertrophy, suggesting that multiple signals are involved in MEF2 activation [94]. Because of the conflicting functions of these two classes of HDACs, the overall effect of HDAC inhibitors on cardiac hypertrophy has been questioned. Several research groups suggest that cardiac hypertrophy can be eliminated by nonspecific or Class I selective HDAC inhibitors [36,95,96]. This phenomenon suggests that the antihypertrophic effect of nonselective HDAC inhibitors is due to the regulation of Class I HDACs. In addition, recent evidence suggests crosstalk between HDAC 2 and class IIa HDACs in the development of cardiac hypertrophy. Acetylation of HDAC 2 preceded phosphorylation, and these modifications were essential for HDAC 2 activation. HDAC 5 regulates the acetylation of HDAC 2. HDAC 2 is one of the important hypertrophy-promoting mediators regulated by class IIa HDACs [97]. One report suggested that HDAC 4 induces hypertension via vascular inflammation and that the administration of TSA dramatically improves high blood pressure [98]. These data suggest that HDACs are a novel therapeutic target for the control of hypertension and resultant cardiac hypertrophy.

### 5.2. Atherosclerosis

Atherosclerosis (AS) is a chronic progressive disease of arteries caused by the abnormal accumulation of lipids, inflammation of multifactorial cells, formation of fibrous capsules, and reactive growth of blood vessels. Various pan- or type-selective HDAC inhibitors have been reported to be prophylactic against AS, which will be discussed below, but the mechanisms by which HDACs are involved in the pathogenesis of AS are so far largely unknown. In one report, it was shown that the suppression of Peroxisome proliferator-activated receptor gamma (PPARγ) expression by HDACs induces foam cell formation of macrophages, leading to the development of AS [99]. Further follow-up studies are needed to reach a final consensus, but it is an interesting finding.

### 5.3. Arrhythmia

In the process of recovery from MI, excessive inflammation and fibrosis in damaged tissue have been shown to lead to an increased incidence of arrhythmias [100]. Thus, the prevention of inflammation is useful in preventing the development of arrhythmias. Up to the present, only a few reports have elucidated the association of HDACs with arrhythmias. TSA dramatically improved atrioventricular conduction abnormalities in the heart of mice induced by the disruption of HopX4 [101]. Considering that HopX directly recruits HDAC 2 and 4, the dramatic effect of TSA could be related to the HopX–HDAC 2 axis [102]. Myocyte-specific knockdown of both HDAC 1 and HDAC 2 results in an abnormal increase in calcium channel subunits [103]. Dysfunction of the calcium channel participates in atrial fibrillation or other types of cardiac arrhythmias, suggesting that altered HDAC activity may cause a variety of arrhythmias [104].

### 5.4. Ischemic Heart Diseases

AS for ischemic heart diseases, it is known that controlling local inflammation after MI is effective in improving the prognosis of treatment, including reducing the incidence of complications. Currently, little is known about how HDACs are involved in the development of inflammation after myocardial infarction. However, there is a significant number of reports suggesting that HDAC inhibitors are effective in improving the prognosis of MI, which will be discussed in the next chapter. In an ex vivo study using the Langendorff system, our group demonstrated that preconditioning TSA preserves cardiac performance after ischemia-reperfusion (I/R) injury [105]. In addition, HDAC inhibitors improve fatty acid oxidation in I/R injury by restoring PGC-1α [106]. HDAC inhibitors are also beneficial for reducing the scar size of myocardial infarction [107]. Up to now, the main beneficial effects of the HDAC inhibitor in I/R injury are believed to be mediated by the inhibition of the generation of immature blood vessels, suppressing inflammation, and promoting energy metabolism.

In addition to the above, HDACs have been implicated in other pathological conditions such as cardiac fibrosis, angiogenesis, and vascular calcification.

### 5.5. Cardiac Fibrosis

Cardiac fibrosis causes a loss of elasticity and inadequate ventricular dilation during the diastole phase, which is considered the primary pathogenesis of heart failure with preserved ejection fraction (HFpEF). HDAC inhibitors dramatically alleviate cardiac fibrosis [95,96]. Alleviation of cardiac fibrosis by HDAC inhibitors may be due to direct action rather than secondary changes after ameliorating cardiac hypertrophy. Furthermore, HDAC inhibitors directly inhibit the differentiation transition from fibroblasts to myofibroblasts, which is regarded as the major pathophysiology of congestive heart failure (CHF) [108]. HDAC 1 and HDAC 2 expression is upregulated in CHF myocardium and treatment with a Class I HDAC inhibitor, mocetinostat, which attenuated the trans differentiation of the fibroblast to prevent the development of interstitial fibrosis [109]. For the past decade, the European society of cardiology and the American Heart Association have issued warnings about the severity of HFpEF [110]. They summarized the clinical outcomes of patients with HFpEF who received conventional regimens for heart failure with reduced ejection fraction (HFrEF). Surprisingly, standard strategies for HFrEF, such as beta-blockers, angiotensin-converting enzyme inhibitors/angiotensin receptor blockers, and aldosterone antagonists, failed to adequately control disease progression in HFpEF. However, given the results using rodent models, HDAC inhibition by HFpEF is the most promising and reproducible strategy for managing HFpEF [111,112]. Future clinical trials are therefore awaited. 

**Figure 3:**
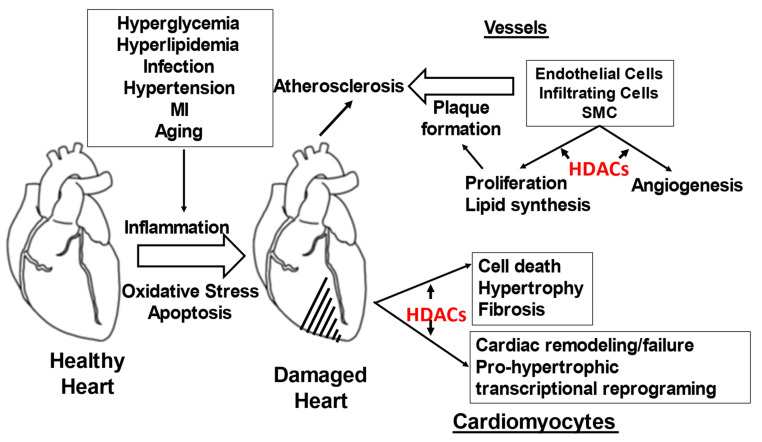
Role of HDACs in various attributes of CVDs pathophysiology.

The role of HDACs in various attributes of CVDs pathophysiology is depicted in Figure 3.

## 6. HDACs as a Therapeutic Target for CVD

**Figure 4:**
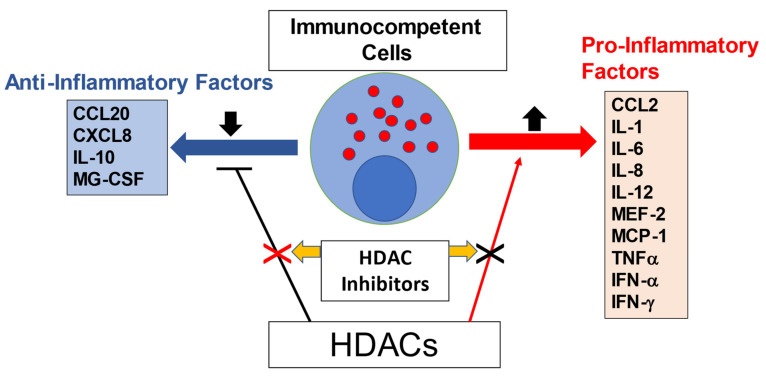
HDAC inhibitor as an anti-inflammatory agent.

Several HDAC inhibitors, along with their anti-inflammatory properties, are gaining attention for their potential as cardiac therapeutic agents (Figure 4). Studies demonstrating the beneficial effects of HDAC inhibitors on CVDs have been conducted intensively over the past decade. We firstly demonstrated a crucial role of HDAC inhibition in protecting the heart against ischemia/reperfusion (I/R) injury in both early and delayed pharmacologic preconditioning following TSA treatments [113]. I/R injury is thought to elicit an innate immune response and therefore it is expected to contribute to increased production of proinflammatory cytokines, including interleukin 1 (IL-1), IL-6, tumor necrosis factor α (TNFα), monocyte chemoattractant protein 1 (MCP-1), and IL-8 by immunocompetent cells [114]. HDAC inhibitors improved cardiac hypertrophy via the inhibition of ROS produced by proinflammatory cytokines [115,116]. In addition, in cultured vascular smooth muscle cells, TNF-induced HDAC 4 mediates vascular inflammation, and TSA treatment dramatically ameliorates the adverse events [117].

Clinical trials for HDAC inhibitors are being conducted primarily in cancer patients. Quit a few investigational HDAC inhibitors have already reached the Phase 3 stage for solid cancer treatment, and several HDAC inhibitors such as Vorinostat, Romidepsin, Belinostat, and Panobinostat have already been approved by the U.S. FDA for the treatment of patients with hematological malignancies [98,118,119,120]. In comparison, clinical trials of HDAC inhibitors for CVD have been somewhat slow. HDACs play an important role in regulating pathogenic signals involved in CVD development, as shown by promising results in various preclinical models. Despite their solid biological background, HDAC inhibitors currently under consideration for the treatment of CVD are very limited, mainly due to their non-selective profiles that cause cardiotoxicity such as QT prolongation. Under these circumstances, hybrid HDAC inhibitors with hydroxy-3-methylglutaryl coenzyme A reductase (HMGR), which would provide an opportunistic pathway for potentially safer and better treatment via dual/multiple inhibitions, are attracting attention. Chen et al. created dual HMGR/HDAC hybrids using lovastatin (HMG-CoA inhibitor) [121]. This hybrid was able to reduce HMGR/HDAC activity yet did not exhibit the high cardiotoxicity of pure-nonspecific HDAC inhibitors and prevented CVDs more safely and effectively. A possible mechanism for this synergistic effect is the direct inhibitory effect of HMGR on HDACs, but the details are not yet known [122]. Currently, there are limited examples of hybrid HDAC inhibitors addressing CVD. However, this strategy is very promising in diseases where pathophysiology and risk factors coexist, such as cancer and/or diabetes-induced cardiomyopathy. Further progress in the clinical application is highly desired.

## 7. Conclusions

In this review, we summarize (1) the pathophysiology of inflammation in cardiovascular disease, (2) the function of HDACs in the regulation of atherosclerosis and cardiovascular diseases, and (3) the possible therapeutic implications of HDAC inhibitors in cardiovascular diseases, with perspective on the relationship between HDAC and inflammation. Many CVDs are derived from atherosclerosis, which is caused by an inflammatory response. Therefore, the CVD suppression effect of HDAC inhibitors may be attributed to their anti-inflammatory effect. Further clarification of the detailed mechanisms will lead to the clinical application of HDAC inhibitors for CVD therapeutics in the near future.

## Data Availability

Not applicable.
